# Sarcomatoid mesothelioma diagnosed in a patient with mesothelioma in situ: a case report on morphologic differences after 9-month interval with details analysis of cytology in early-stage mesothelioma

**DOI:** 10.1186/s13000-023-01416-7

**Published:** 2023-11-28

**Authors:** Miho Yoshida, Naoe Jimbo, Ryuko Tsukamoto, Tomoo Itoh, Kunimitsu Kawahara, Suguru Mitsui, Yugo Tanaka, Yoshimasa Maniwa

**Affiliations:** 1 Department of Diagnostic Pathology, Kobe University Hospital, Address: 7-5-2 Kusunoki-Cho, Chuo-Ku, Kobe, 650-0017 Japan; 2 Division of Pathology for Regional Communication, Kobe University Graduate School of Medicine, Address: 7-5-2 Kusunoki-Cho, Chuo-Ku, Kobe, 650-0017 Japan; 3 Division of Thoracic Surgery, Kobe University Graduate School of Medicine, Address: 7-5-2 Kusunoki-Cho, Chuo-Ku, Kobe, 650-0017 Japan

**Keywords:** CDKN2A, Cell-in-cell engulfment, Mesothelioma in situ, MTAP, Sarcomatoid mesothelioma

## Abstract

**Background:**

Overlapping morphological features of mesothelial cells have been rendered it difficult to distinguish between reactive and malignant conditions. The development of methods based on detecting genomic abnormalities using immunohistochemistry and fluorescence in situ hybridization have contributed markedly to solving this problem. It is important to identify bland mesothelioma cells on cytological screening, perform efficient genomic-based testing, and diagnose mesothelioma, because the first clinical manifestation of pleural mesothelioma is pleural effusion, which is the first sample available for pathological diagnosis. However, certain diagnostic aspects remain challenging even for experts.

**Case presentation:**

This report describes a case of a 72-year-old man with a history of asbestos exposure who presented with pleural effusion as the first symptom and was eventually diagnosed as mesothelioma. Mesothelioma was suspected owing to prominent cell-in-cell engulfment in mesothelial cells on the first cytological sample, and the diagnosis of mesothelioma in situ was confirmed by histology. Unexpectedly, sarcomatoid morphology of mesothelioma was found in the second pathology samples 9 months after the first pathological examination. Both the mesothelioma in situ and invasive lesion showed immunohistochemical loss of methylthioadenosine phosphorylase (MTAP) and homozygous deletion of cyclin dependent kinase inhibitor 2A (*CDKN2A*) on fluorescence in situ hybridization*.* The patient received medication therapy but died of disease progression 12 months after the diagnosis of the sarcomatoid morphology of mesothelioma.

**Conclusion:**

Our case suggests that cell-in-cell engulfment can be conspicuous in early-stage mesothelioma with inconspicuous nuclear atypia and few multinucleated cells. In addition, the presence of MTAP loss and *CDKN2A* homozygous deletion are suspected to be involved in early formation to invasive lesions and/or sarcomatoid morphology. We believe that it is important to consider genetic abnormalities when deciding on individual patient management. Furthermore, cases of mesothelioma, even those of an in situ lesion, with MTAP loss and/or *CDKN2A* deletion should be carefully followed up or subjected to early treatment.

## Background

Pleural mesothelioma is a rare malignant tumor with a poor prognosis, and the incidence of pleural mesothelioma is increasing in some countries [1].The first clinical manifestation of pleural mesothelioma is pleural effusion, which comprises the first specimen for cytological diagnosis. Historically, it has been difficult to distinguish between reactive and malignant mesothelial cells owing to their overlapping morphological characteristics [1–3]. However, the development of methods based on detecting the genomic abnormalities using immunohistochemistry (IHC) and fluorescence in situ hybridization (FISH) have contributed greatly to solve this problem. When mesothelioma cells show no overt cytological atypia in body cavity effusions, these cells are likely to be treated as reactive cells and may not proceed to the following step. However, it is important to identify bland mesothelioma cells on cytological screening and subject these cases to efficient IHC and/or FISH. Here, we report a notable case of the sarcomatoid morphology of invasive mesothelioma that first developed as mesothelioma in situ (MIS). One of the significant aspects noted herein is that both the MIS and invasive mesothelioma exhibited loss of methylthioadenosine phosphorylase (MTAP) and homozygous deletion of cyclin-dependent kinase inhibitor 2A (*CDKN2A*). These abnormalities were the same as those found in the case of sarcomatoid mesothelioma arising from MIS previously reported by our group [4, 5]. Another important finding reported herein is the prominent cell-in-cell engulfment evidenced in the pleural effusion cytology of the first pathology examination. The cell-in-cell engulfment, also known as cell-within-cell arrangements or cell-in-cell invasion, is a cytomorphological finding indicating mesothelioma [6–8]. Our case showed the possibility of a relatively high frequency of cell-in-cell engulfment appearing in the body cavity effusion in mesothelioma at an early stage without noticeable cytological atypia or multinucleated cells.

## Case presentation

**Fig. 1:**
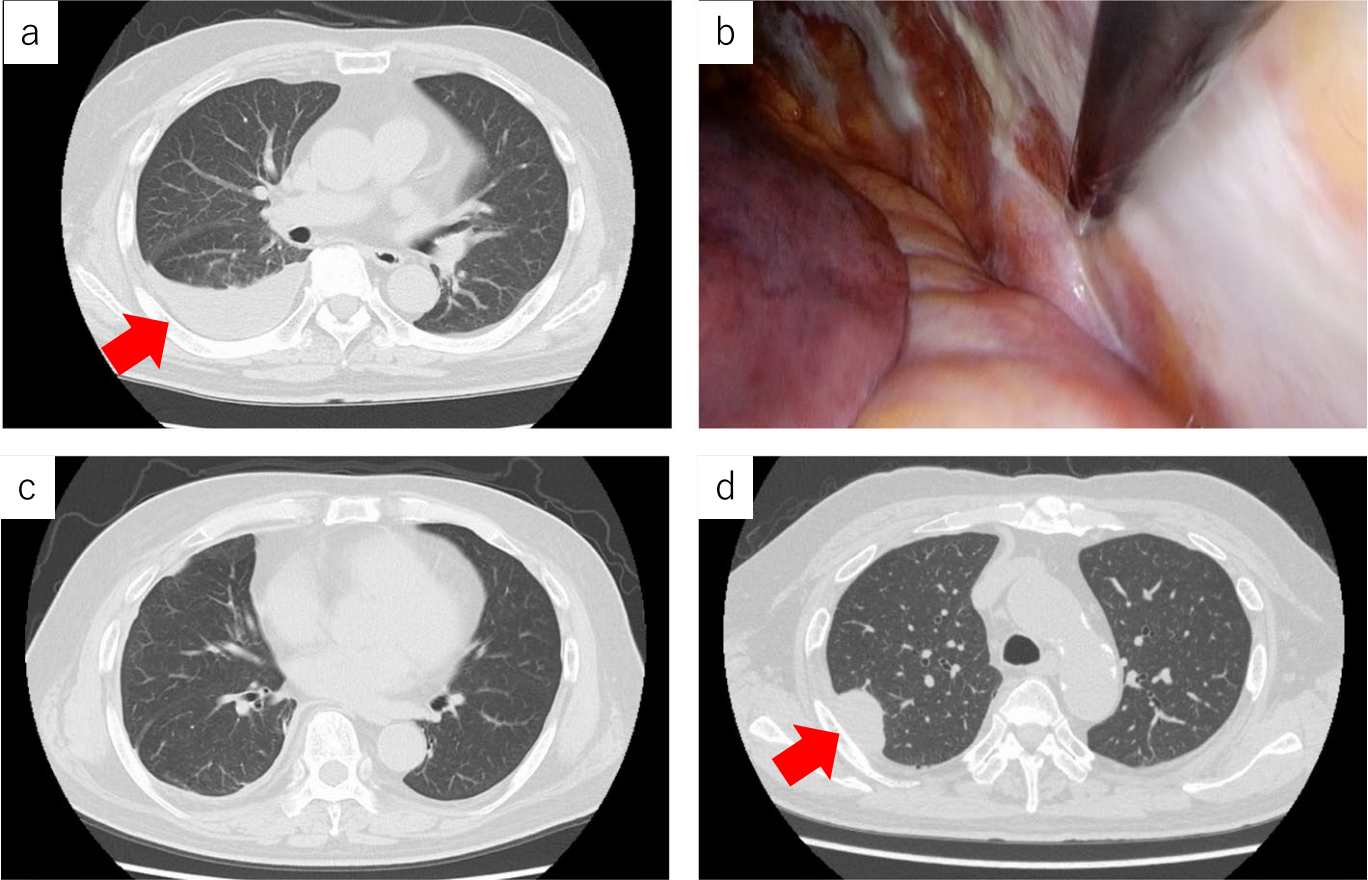
Clinical images. **a** Chest computed tomography showed a moderate amount of right pleural effusion with a slight bilateral pleural thickening. **b** A video-assisted thoracoscopic surgery biopsy of the right parietal pleura showed plaque-like pleural thickening, but no visible tumor. **c** The right pleural effusion decreased within five months from the first biopsy. **d** A nodular tumor approximately 3 cm in size appeared in the right pleura nine months from the first biopsy

A 72-year-old man, who was a heavy smoker (50 pack-year), underwent treatment for right pleural effusion at a different hospital. He had a history of occupational asbestos exposure as a welder. His past medical history included lung emphysema, diabetes, hypertension, hyperlipidemia, and spinal canal stenosis. Eleven months after the initial onset of pleural effusion, he experienced recurrent effusion and was referred to our hospital. Chest computed tomography (CT) showed a moderate amount of right pleural effusion (Fig. 1a). Thoracoscopically, multiple plaques and pleural effusion (600 mL in volume) were detected, but no visible tumors were identified (Fig. 1b). A right pleural biopsy assisted by video-assisted thoracic surgery (VATS) led to the diagnosis of MIS. We continued careful follow-up.

The pleural effusion decreased after 5 months from the first biopsy (Fig. 1c). However, a solid nodule appeared in the right parietal pleura at 9 months post-biopsy (Fig. 1d). A second histological examination (CT-guided biopsy) showed spindle cells without any epithelioid atypical cells, with clear invasion. The patient underwent immunotherapy with ipilimumab and nivolumab followed by combination chemotherapy (including carboplatin and pemetrexed). However, the target lesions were clinically evaluated as a progressive disease during treatment. The patient died 33 months after the onset of the first symptoms of pleural effusion (21 months after the first biopsy and 12 months after the second biopsy).

### Pathological findings

**Fig. 2:**
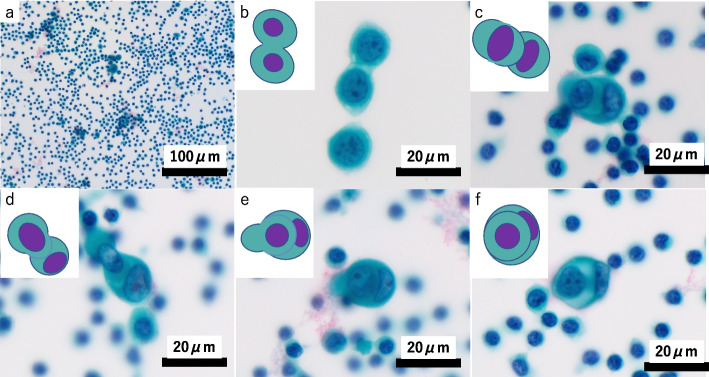
Cytological findings of cytocentrifuge smear specimen of the fist cytological sample (right pleural effusion cytology). **a** Mesothelial cells were scattered and were accompanied by severe inflammatory cells. **b** Cell-to-cell apposition were also seen. Cell-in-cell engulfment including cytoplasmic inclusion (**c**), nuclear inclusion (**d**) typical molded cells with hump-like cytoplasmic processes (**e**), and paired-cells (**f**), were evidenced

**Fig. 3:**
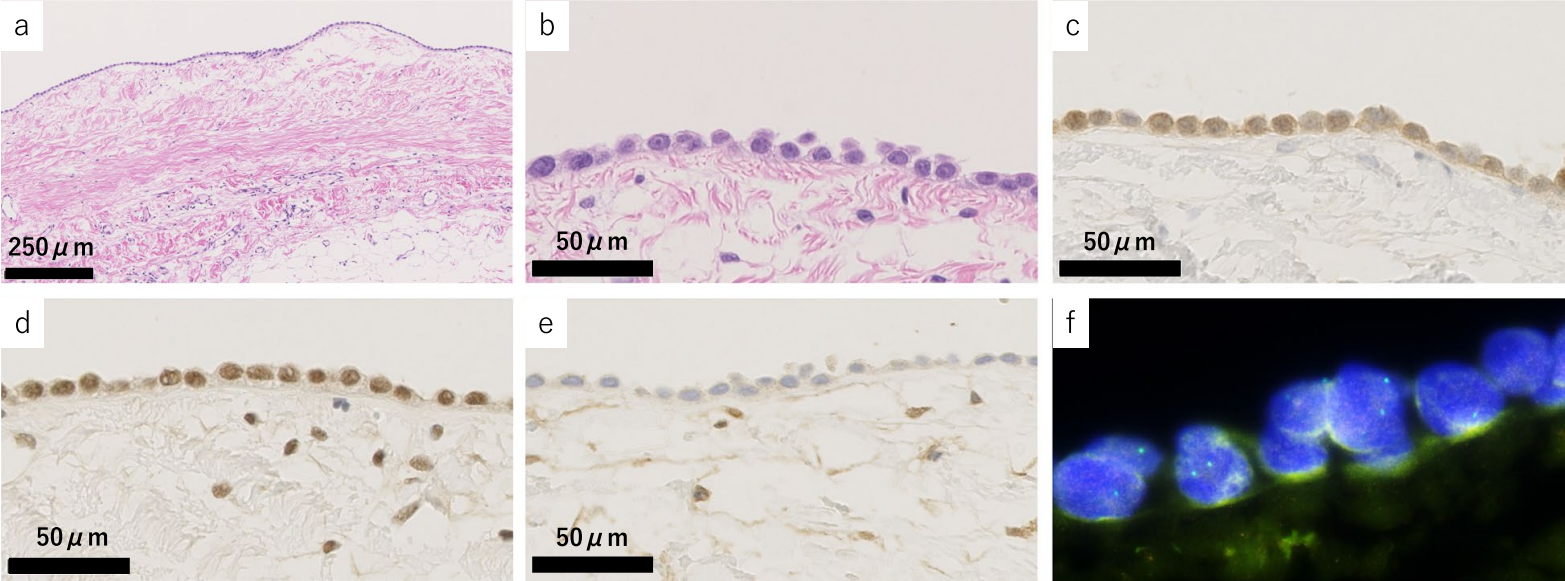
Histological findings from the first sample (video-assisted thoracoscopic surgery biopsy for right pleura) (**a**–**f**). **a**, **b** A single layer of proliferating mesothelial cells with atypia was detected. **c** These mesothelial cells were positive for pan-cytokeratin (CAM5.2). **d** BAP1 was retained. **e** MTAP was lost in focal areas of the surface mesothelium. **f** Homozygous deletion of *CDKN2A* (61.5%) was detected in the surface mesothelium by fluorescence in situ hybridization (*CDKN2A* probe, red label; chromosome 9 centromeric probe, green label)

The first pathological examination included right-side pleural fluid cytology and a right-side parietal pleural biopsy with VATS. In the pleural-fluid cytology sample, 1,201 mesothelial cells per cytocentrifuge smear specimen made from 6 mL were found, which was much lower than that evidenced in the overt mesothelioma in our department (over 10,000 cells on average in 3 cases). The mesothelial cells were scattered in the inflammatory cells, with occasional cell-to-cell apposition (Fig. 2a, b). The mesothelial cells were mainly mononuclear. Binucleated cells and multinucleated mesothelial cells with 3–5 nuclei were scarce (4.0% and 1.2%, respectively). The nuclear-cytoplasm ratio was low and nuclear atypia was mild. These findings were inconsistent with the typical pathological observations of mesothelioma. However, cell-in-cell engulfment with or without hump-like cytoplasmic processes and/or paired cells (Fig. 2c–f) were conspicuous in 7.3% of the evaluated cells. The pleural biopsy assisted by VATS was performed from five different sites. Histologically, a single layer of mesothelial cells with atypia spread along the surface of the pleura (Fig. 3a, b). The mesothelial cells were positive for mesothelial markers such as calretinin (DAK Calret1, 1:15; Dako, Glostrup, Denmark), D2-40 (D2-40, 1:100; Dako), WT-1 (WT49, 1:40; Leica Biosystems, Wetzlar, Germany), HEG1 (SKM9-2, prediluted; Nichirei Biosciences, Inc., Tokyo, Japan), and pan-cytokeratin (CAM5.2, 1:10; BD Biosciences, Haryana, India) (Fig. 3c). The mesothelial cells were negative for carcinoma markers, including Ber-EP4 (Ber-EP4, prediluted; Dako), MOC31 (MOC-31, 1:60; Dako), and Claudin 4 (3E2C1, 1:200; Invitrogen, Waltham, MA, USA). All mesothelial cells retained BAP1 expression (C-4 1:100, Santa Cruz Biotechnology) (Fig. 3d), while some mesothelial cells lost MTAP expression (2G4, 1:200, Abnova Corporation) (Fig. 3e). In the areas where MTAP expression was lost, 61.5% (115/187) of the mesothelial cells showed homozygous deletion of *CDKN2A* on FISH (Agilent Technologies, Santa Clara, CA, USA) (*CDKN2A* probe, red label; chromosome 9 centromeric probe, green label) (Fig. 3f). Based on these findings and multidisciplinary discussion, this pleural lesion was concluded to be MIS.

**Fig. 4:**
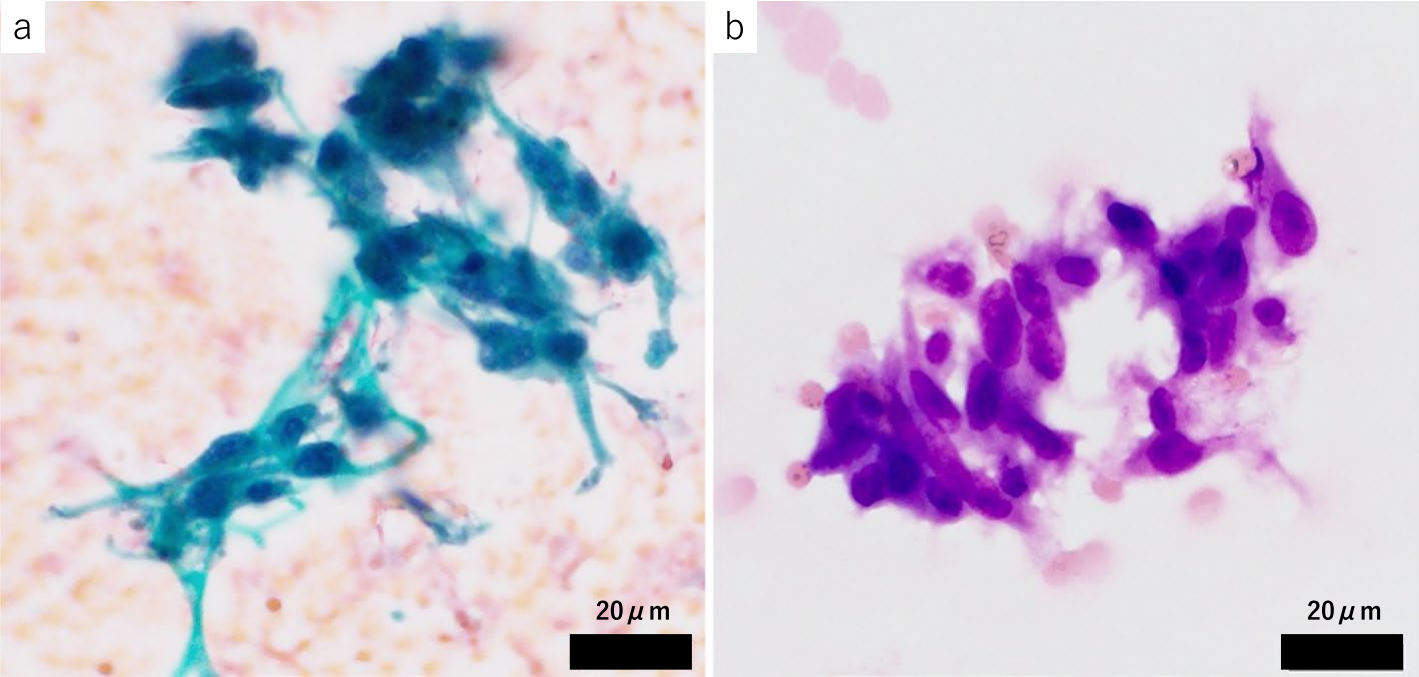
Cytological findings of the washing cytology sample of needle-washing cytology used in biopsy (**a**, **b**). Spindle-shaped and loosely connective atypical cells appeared with necrosis in Papanicolaou stain (**a**) and Giemsa stain (**b**)

**Fig. 5:**
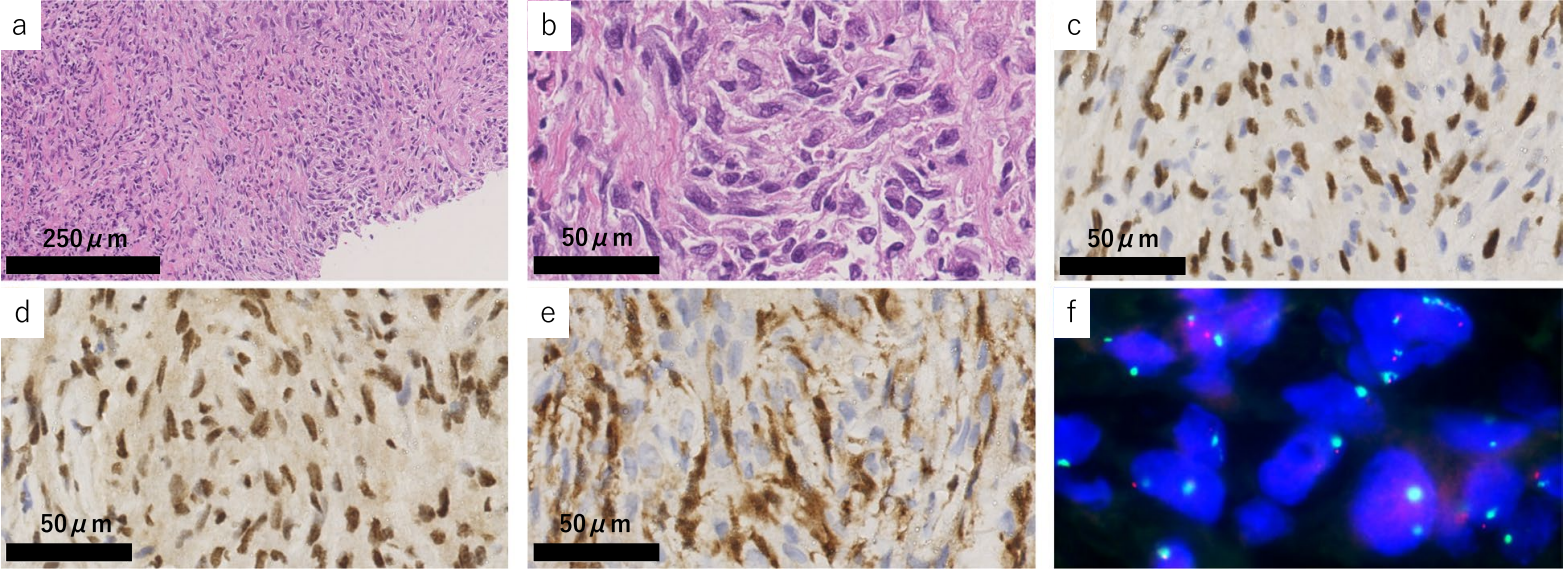
Histological findings from the second sample (computed tomography-guided biopsy for a solid nodule in right pleura) (**a**–**f**). **a**, **b** Spindle-shaped tumor cells with strong nuclear atypia and nuclear disparities in size show diffused growth with loose connectivity. **c** WT1 was focal positive. **d** BAP1 was retained. **e** MTAP was absent in almost all tumor cells. **f** Homozygous deletion of *CDKN2A* (31.0%) was confirmed in tumor areas

The second pathology samples included a CT-guided biopsy tissue sample and needle-washing cytology used in the CT-guided biopsy. The needle-washing cytology showed atypical spindle cells in the necrotic debris (Fig. 4a, b). Histologically, the tumor was composed of atypical spindle cells arranged in abortive fascicles within the fibrous stroma (Fig. 5a, b). The atypical cells were positive for pan-cytokeratin. The cells were weakly or focally positive for WT1 (Fig. 5c) and HEG1 and were negative for calretinin and D2-40. Atypical cells were positive for BAP1 (Fig. 5d), while most atypical cells lost cytoplasmic MTAP expression (Fig. 5e). At least 31% (31/100) of mesothelial cells showed homozygous deletion of *CDKN2A* on FISH (Fig. 5f). These finding are consistent with sarcomatoid mesothelioma cells. There was a possibility of biphasic mesothelioma, but the absence of epithelioid atypical cells in either needle-washing cytology sample or histological sample highly suggested the possibility of sarcomatoid mesothelioma.

## Discussion and conclusions

To our knowledge, the case reported herein is the second case of sarcomatoid morphology of mesothelioma that first developed as MIS, following the first reported case—which was also presented by our research group [4, 5]. Notably, both cases shared the same genomic abnormalities (BAP1 retain, MTAP loss, and *CDKN2A* homozygous deletion). The presence of MTAP loss and *CDKN2A* homozygous deletion are suspected to be involved in the early formation of invasive lesions and/or sarcomatoid morphology.

MIS was first proposed in 1992 [9]. However, until recently, it had been unclear if there was truly an in situ state. The concept of MIS has recently become accepted with the development of genome-based diagnostic methods [10]. BAP1 IHC, MTAP IHC, and *CDKN2A* FISH are gold-standard methods for distinguishing malignant mesothelioma from benign mesothelial lesions, with high sensitivity (80–90% in combination) and 100% specificity [11]. BAP1 loss is detected more in epithelioid mesothelioma (60–70%) than in sarcomatoid mesothelioma (< 40%), while MTAP loss and *CDKN2A* homozygous deletions are detected more in sarcomatoid mesothelioma (~ 90%) than in epithelioid mesothelioma (60–70%) [11, 12]. High homozygous deletion of *CDKN2A* in pleural mesothelioma correlates with poor prognosis [13], which may be associated with a high proportion of sarcomatoid features in MTAP-loss mesothelioma.

Although attention towards MIS has increased, indications for and timing of MIS treatment are under discussion. Klebe et al. reported that a majority of MIS patients had received only follow-up, while the remaining patients received active treatment [14]. These researchers also reported variation in the time of progression from MIS; 15% of the MIS cases progressed to invasive mesothelioma within 6–12 months, while 35% showed progression ≥ 4 years later [14]. We speculate that one reason for this variation may be due to genetic abnormalities. We believe that careful follow-up or early intervention can be crucial for MIS patients, at least those with MTAP loss and/or *CDKN2A* homozygous deletions.

Cell-in-cell engulfment, also known as cell-within-cell arrangements or cell-in-cell invasion, is one form of the nonapoptotic cell death process in matrix-detached cells [15]. Cell-in-cell engulfment is distinguished from cell-to-cell apposition, which is adhesion; cell-to-cell apposition is found in stimulated mesothelial cells, whether malignant or benign, while cell-in-cell engulfment is more frequently observed in mesothelioma [7, 8]. Pleural mesothelioma cells with 9p21 homozygous deletion have been reported to show significantly higher frequent cell-in-cell engulfment than benign reactive mesothelial cells (mean, 17.4–20.6% for mesothelioma, 3.5–4.1% for benign reactive mesothelial cells) [7, 8]. The percentage of cell-in-cell engulfment in the first cytological sample of our case was 7.3%, which was much lower than that in mesothelioma but almost twice as frequent as that in benign reactive mesothelial cells [7, 8]. Cell-in-cell engulfment is infrequently seen in histological samples, suggesting that this pattern occurs during or after exfoliation of tumor cells into the body cavity fluid [2]. The International Mesothelioma Interest Group guidelines suggest the following nine cytomorphological criteria indicating mesothelioma, which includes cell-in-cell engulfment as a cell-within-cell arrangement: 1) high cellularity, 2) large size, 3) papillary clusters, 4) acidophilic matrix, 5) macronucleoli, 6) protrusion from the cell membrane, 7) prominent degree of cell-within-cell arrangement, 8) multinucleated cells, and 9) vacuoles overlapping the nuclei on Giemsa staining [6]. Although it is challenging to suspect malignant mesothelioma because cytological findings of our first cytology sample met few of these criteria and were not typical of invasive mesothelioma, our case shows that cell-in-cell engulfment may be prominent in the cytomorphology of early-stage mesothelioma. This also suggested the necessity of paying attention to not only overt cytomorphological findings but also cell-in-cell engulfment, because this might be observed from the very early stages of mesothelioma.

Our study has a few limitations. First, there is always a limitation in regard to the representativity of the samples, especially in the second biopsy, where cytology was also performed using the washing of the needle used for the biopsy (which explains the same cell population). Because of this sampling issue, a biphasic mesothelioma can not be completely ruled out. A great opportunity to be able to do proper sampling would have been an autopsy, but this was unfortunately not performed. Second, whether MIS really progressed to this tumor consisting of sarcomatoid or spindle cells can only be proven with a clonal-evolution type of genomic analysis. However, it is noteworthy and interesting that sarcomatoid tumor cells occurred 9 months after the initial MIS diagnosis.

In conclusion, MTAP loss and/or *CDKN2A* homozygous deletion in MIS may indicate aggressive features, based on our two MIS cases with MTAP loss and *CDKN2A* homozygous deletions that ultimately showed invasive mesothelioma with sarcomatoid morphology. Re-biopsy of invasive lesions after diagnosing MIS is essential, especially for MIS presenting with these types of genetic abnormalities. When the number of mesothelial cells with cell-in-cell engulfment is high, the possibility of mesothelioma should be considered, even if there are no overt atypical cytological findings on effusion cytology.

## Data Availability

Data sharing is not applicable to this article as no datasets were generated or analyzed in the reporting of this case.

## Abbreviations

IHC: Immunohistochemistry
FISH: Fluorescence in situ hybridization
MIS: Mesothelioma in situ
MTAP: Methylthioadenosine phosphorylase
CDKN2A: Cyclin dependent kinase inhibitor 2A
CT: Computed tomography
VATS: Video-assisted thoracic surgery

## References

[1] Eccher A, Girolami I, Lucenteforte E, Troncone G, Scarpa A, Pantanowitz L (2021). Diagnostic mesothelioma biomarkers in effusion cytology. Cancer Cytopathol.

[2] Hamakawa S, Mori K, Kashiwazaki Y, Tanabe M, Kondo Y, Sakamaki K (2003). Molded mesothelial cells with hump-like cytoplasmic process in effusion cytology of malignant mesothelioma. J Jpn Soc Clin Cytol.

[3] Haefliger S, Savice Prince S, Rebetez J, Borer H, Bubendorf L (2021). Putative malignant pleural mesothelioma in situ (MPMIS) with sequential acquisition of genomic alterations on fluorescence in situ hybridization (FISH) examination. Acta Cytol.

[4] Minami K, Jimbo N, Tanaka Y, Hokka D, Miyamoto Y, Itoh T (2020). Malignant mesothelioma in situ diagnosed by methylthioadenosine phosphorylase loss and homozygous deletion of CDKN2A: a case report. Virchows Arch.

[5] Nishikubo M, Jimbo N, Tanaka Y, Tachihara M, Itoh T, Maniwa Y (2022). Sarcomatoid mesothelioma originating from mesothelioma in situ: are methylthioadenosine phosphorylase loss and CDKN2A homozygous deletion poor prognostic factors for preinvasive mesothelioma?. Virchows Arch.

[6] Hjerpe A, Ascoli V, Bedrossian CWM, Boon ME, Creaney J, Davidson B, et al. Guidelines for the cytopathologic diagnosis of epithelioid and mixed-type malignant mesothelioma. Complementary statement from the International Mesothelioma Interest Group, also endorsed by the International Academy of Cytology and the Papanicolaou Society of Cytopathology. Acta Cytol. 2015;59:2–16. 10.1159/000377697 10.1159/000377697 25824655

[7] Matsumoto S, Nabeshima K, Kamei T, Hiroshima K, Kawahara K, Hata S (2013). Morphology of 9p21 homozygous deletion-positive pleural mesothelioma cells analyzed using fluorescence in situ hybridization and virtual microscope system in effusion cytology. Cancer Cytopathol.

[8] Matsumoto S, Hamasaki M, Kinoshita Y, Kamei T, Kawahara K, Nabeshima K (2019). Morphological difference between pleural mesothelioma cells in effusion smears with either BAP1 loss or 9p21 homozygous deletion and reactive mesothelial cells without the gene alterations. Pathol Int.

[9] Whitaker D, Henderson DW, Shilkin KB (1992). The concept of mesothelioma in situ: implications for diagnosis and histogenesis. Semin Diagn Pathol.

[10] Churg A, Galateau-Salle F, Roden AC, Attanoos R, von der Thusen JH, Tsao MS (2020). Malignant mesothelioma in situ: morphologic features and clinical outcome. Mod Pathol.

[11] Churg A, Naso JR. The separation of benign and malignant mesothelial proliferations: new markers and how to use them. Am. J. Surg. Pathol. 2020;44:e100–12). 10.1097/PAS.0000000000001565 10.1097/PAS.0000000000001565 32826526

[12] Kinoshita Y, Hida T, Hamasaki M, Matsumoto S, Sato A, Tsujimura T (2018). A combination of MTAP and BAP1 immunohistochemistry in pleural effusion cytology for the diagnosis of mesothelioma. Cancer Cytopathol.

[13] Hamasaki M, Matsumoto S, Abe S, Hamatake D, Kamei T, Hiroshima K (2016). Low homozygous/high heterozygous deletion status by p16 FISH correlates with a better prognostic group than high homozygous deletion status in malignant pleural mesothelioma. Lung Cancer.

[14] Klebe S, Nakatani Y, Dobra K, Butnor KJ, Roden AC, Nicholson AG (2021). The concept of mesothelioma in situ, with consideration of its potential impact on cytology diagnosis. Pathology.

[15] Overholtzer M, Mailleux AA, Mouneimne G, Normand G, Schnitt SJ, King RW (2007). A nonapoptotic cell death process, entosis, that occurs by cell-in-cell invasion. Cell.

